# Care Integration in Primary Dementia Care Networks: A Longitudinal Mixed-Methods Study

**DOI:** 10.5334/ijic.5675

**Published:** 2021-12-08

**Authors:** Dorien L. Oostra, Anne Harmsen, Minke S. Nieuwboer, Marcel G. M. Olde Rikkert, Marieke Perry

**Affiliations:** 1 Radboud university medical center, Radboud Institute for Health Sciences, Department of Geriatric Medicine, Nijmegen, NL; 2 Radboudumc Alzheimer Center, Radboud university medical center, Nijmegen, NL; 3 HAN University of Applied Sciences, Academy of Health and Vitality, Nijmegen, NL; 4 Radboud university medical center, Donders Institute for Brain Cognition and Behaviour, Department of Geriatric Medicine, Nijmegen, NL; 5 Radboud university medical center, Donders Institute for Brain Cognition and Behaviour, Department of Primary and Community Care, Nijmegen, NL

**Keywords:** network-based care, dementia care, primary care, interprofessional collaboration, integrated care

## Abstract

**Introduction:**

Currently, care integration for community-dwelling persons with dementia is poor and knowledge on how to effectively facilitate development of integrated dementia care is lacking. The DementiaNet program aims to overcome this with a focus on interprofessional collaboration. The objective of this study is to investigate how care integration in interprofessional primary dementia care networks matures and to identify factors associated with (un)successfully maturation.

**Theory and methods:**

A longitudinal mixed-methods study, including 17 primary care networks participating in the DementiaNet study, was performed. Semi-structured interviews based on the Rainbow Model of Integrated Care were conducted at start, at 12- and 24 months. Network maturity scores (range 1–4) were derived from the interviews and qualitative data was used to explain the observed patterns.

**Results:**

Networks consisted on average of 9 professionals (range 4–22) covering medical, care and social disciplines. Network maturity yearly increased with 0.29 (95%-CI: 0.20–0.38). Important factors for improvement included getting to know each other’s expertise, having a capable network leader(s), stable network composition and participation of a general practitioner.

**Conclusions:**

The DementiaNet approach enables a transition towards more mature networks. Identified success factors provide better understanding of how network maturity can be achieved and gives guidance to future care integration strategies.

## Introduction

The rapid ageing population together with the rising number of older adults with chronic conditions creates a major challenge for healthcare systems [1]. In the Netherlands, many older adults with dementia remain living at home, due to policy changes that led to closing of elderly homes. Consequently, the burden on primary care is increasing. Dementia is a condition that affects multiple aspects of the lives of persons with dementia and their caregivers. Especially in later stages of the disease, several professionals of medical, care and social disciplines are involved. Often these professionals work at different organisations, and fragmentation of care is likely to arise [2]. As a result, continuity of care is lacking and there is a low satisfaction with the provided care among professionals, persons with dementia and their informal caregivers [3,4].

Integrated primary care is considered a strategy to overcome care fragmentation, by increasing the collaboration between professionals and care organizations thereby improving the healthcare system’s continuity of care [5,6,7,8]. Integrated care is a complex term and different terminologies are used to describe this concept [9]. The WHO defines integrated care as the delivery of a continuum of care, designed to meet multidimensional needs of the population and the individual, by a coordinated multidisciplinary team of professionals [10]. To achieve integrated care, a transition towards network-based care is suggested [6,11,12]. However, empirical evidence is still lacking [13], which is essential for implementing such network activities in dementia care.

The DementiaNet approach brought the transition towards network-based care into practice. DementiaNet is designed to facilitate the development of interprofessional networks of primary care professionals from the medical, health and social care services [11,14]. As in other transitions, sufficient time is needed for collaborations to mature and achieve care integration. Support from the DementiaNet team is therefore provided for a period of two years. However, it is unclear whether this intentional transition leads to improvement of care integration.

We aim to investigate how the DementiaNet approach affects network maturity of these interprofessional primary dementia care networks over time. Additionally, we will identify factors associated with (un)successful network maturation.

## Theory and Methods

### Study design

A longitudinal multiple case mixed-method study was performed to evaluate the development of network maturity of networks participating in the DementiaNet program. We chose a mixed-method design, applying and integrating quantitative and qualitative data sources, to gain deeper understanding of the mechanisms behind (un)successful network maturation [15,16]. Each DementiaNet network served as a case with a 24 months follow-up. This study was conducted within the Dutch primary dementia care setting. Detailed description of primary care in the Netherlands can be found in Appendix I.

The study protocol was submitted for review to the local ethical committee, and they declared that formal judgment was not required according to the Dutch law (protocol number: 2019–5599).

### Study population

New and existing local collaborations of primary care professionals, with a shared caseload of dementia patients, could voluntarily participate in the DementiaNet program. Composition of the networks was tailored to local availability and preferences. Consequently, each network was different in terms of size and represented disciplines and starting levels of collaboration and quality of care. Desirably, networks included at least one professional of the medical (e.g. general practitioner), care (e.g. community nurse) and social (e.g. social worker or case manager) discipline. The networks started with the DementiaNet program between 2015 and 2017 [11]. All participating networks were located in the east of the Netherlands [17].

### DementiaNet program

**Figure 1:**
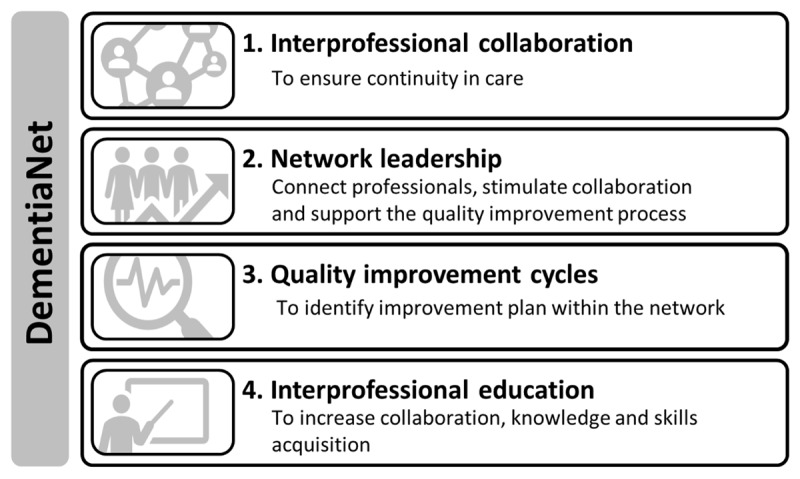
Key elements of the DementiaNet program.

The DementiaNet program is a stepwise, bottom-up approach to facilitate integrated care implementation. The DementiaNet program consists of four key elements (see ***Figure 1***) aimed at achieving networks to become independent, sustainable and interprofessional collaboratives, in which members can provide better quality of care and achieve higher effectiveness.

The first element of the DementiaNet program is interprofessional collaboration. The networks of professionals, offering services to a shared caseload of people with dementia, should want to achieve structured local interprofessional collaboration to ensure continuity in care.

Second, at least one network participant is the network leader and is trained and coached by the DementiaNet team to support them in their leadership role. The key tasks of the network leader include: connect professionals, stimulate collaboration and support the quality improvement process.

Third, networks should engage in quality improvement cycles to improve the quality of care. At least once a year an improvement plan is carried out based on their yearly quality of care assessment and benchmark feedback. Based on these results the network members jointly identify improvement goals.

Fourth, interprofessional education about self-selected topics is offered to the networks to increase collaboration, knowledge and skills acquisition. The contents of these training and coaching sessions are tailored to each network’s own goals.

The elements of the DementiaNet program are tailored to the needs of the networks, thereby allowing for the large practice variation present in daily clinical practice. Networks are supported by the DementiaNet project team during a period of two years.

More detailed information about the DementiaNet program is described in Appendix II and elsewhere [14].

### Theoretical framework

**Figure 2:**
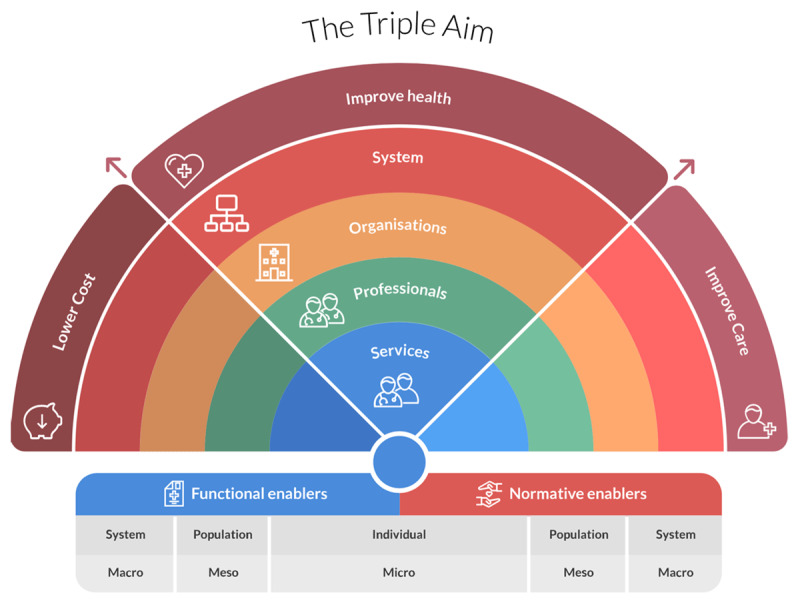
Rainbow Model of Integrated Care. Adapted with permission from Essenburgh Research & Consultancy [22].

Theoretical frameworks were used to asses network maturity towards integrated care, which we define as “*a coordinated way of working across multiple professionals, organisations and sectors in order to improve the health, quality of care and economic outcomes for a targeted (sub)population*” [18]. During the development of the DementiaNet approach (2014), we focused on interprofessional collaboration and network development, which was, at that time, still a novel approach. We used the collaborative network theory of Kaats and Opheij [19] as a foundation. The Rainbow Model of Integrated Care (RMIC), developed in the same period, provided a theoretical framework for integrated care [20] and had important parallels with the DementiaNet approach since the programs’ core elements were all represented in the model (e.g. professional integration, leadership and quality improvement). The RMIC is a validated framework that emphasizes the complexity of integrated primary care and defines key elements for achieving it [21]. Different integration domains are specified in the RMIC, therefore it is possible to identify areas for improvement. The RMIC describes three categories of integrated care: the scope, type and enablers of integration, including 8 domains (see ***Figure 2***). The DementiaNet approach includes most RMIC domains.

#### Scope of integration

The scope is the person and population focused view of professionals, e.g. focusing on patient’s needs and abilities instead of the disease (person-focused care) and meeting a target groups’ specific healthcare requirements (population-focused care).

Both person-focused and population-focused care are incorporated in the DementiaNet program. The overall aim of DementiaNet is to improve person-focused care for people with dementia and their caregiver(s). Networks are stimulated to, for example, consult the person with dementia and their caregiver before a multidisciplinary meeting or to talk about future care wishes. Additionally, DementiaNet aims for networks to identify the population with dementia better, for example, by earlier recognition of signs of cognitive deterioration.

#### Type of integration

The type of integration consists of integration on the micro (individual), meso (population) and macro (system) level. The type of integration refers to four domains: 1) delivered and coordinated services to patients (clinical integration), 2) collaboration between healthcare professionals (professional integration), 3) collaboration between healthcare organizations (organizational integration) and 4) implementation of new policies and regulations (system integration). The network leader facilitates collaborations between and within the micro, meso and macro level. The DementiaNet program mainly focuses on clinical and professional integration. Network members are encouraged to share their tasks and expertise, thereby getting to know each other’s expertise. Moreover, they are stimulated to schedule frequent multidisciplinary meetings and implement multidisciplinary care plans for their shared caseload. The networks are encouraged to not only coordinate the care for their shared patients but also make work agreements about the care for the entire population.

#### Enablers of integration

Functional and normative enablers are needed to establish connectivity between the micro, meso and macro level. Functional enablers are for example communication tools that can be used by all the professionals and organizations in a network. Normative enablers refer to the development and maintenance of a common goal or plans for improvement. The DementiaNet program actively facilitates multiple enablers such as leadership, quality improvement cycles, interprofessional education, discussing the shared vision and implementing digital communication tools.

As described above these eight integration domains, divided in three categories are almost all incorporated in our DementiaNet approach. Currently, a validated integrated care measurement tool, considering the different levels of care provision, is lacking [23,24,25,26,27]. Therefore, during the start of DementiaNet program, we considered the RMIC domains the best option to assess the network maturation towards integrated care of these networks. We developed a scorings system based on the conceptual representation of network maturity at four different maturity levels ad hoc, defined, controlled and synchronized collaboration from qualitative data. Thereby, we were able to assess the collaboration between care professions as a network and identify improvement areas for practice and research [28,29,30]. Detailed information about the protocol for evaluation of DementiaNet can be found elsewhere [11].

### Data collection and measurements

#### Network maturity

To assess network maturity, yearly semi-structured face-to-face interviews were conducted by trained researchers (IM or DO) with the network leader(s). Network maturity was assessed at three timepoints (T0, T1 and T2) by conducting an interview at 12 and 24 months. During the first interview at 12 months we combined the baseline (T0) and 12 months (T1) data-collection by determining the differences between the current situation and before they started with the DementiaNet program. Data was collected between January 2015 and June 2019. A topic list was used (Appendix III), based on the eight domains of the RMIC. Interviews were audio recorded, the length of the interviews varied between 20 and 60 minutes. Network leaders gave written informed consent prior to the interview.

A network’s maturity was defined by rating the interviews for the eight domains of the RMIC. A scale with four predefined levels was used: 1 = ad hoc, 2 = defined, 3 = controlled and 4 = synchronized collaboration. Scores ranged from 1–4 (including half points) and a higher score indicated higher network maturity. Rating was performed independently by two researchers (DO, AH), using an elaborate protocol (on request available) to standardize the scoring after which consensus was reached on each item. A total network maturity score and sub scores eight domains of the RMIC were calculated.

#### Network characteristics

The researchers documented the network characteristics at start, changes in network composition, network leaders, and their leadership abilities (***Table 1***).

**Table 1:** Characteristics of the DementiaNet primary dementia care networks.


NETWORK	DISCIPLINES INVOLVED	DISCIPLINES INVOLVED	NETWORK LEADER(S)	NETWORK LEADER(S) CHANGED	NETWORK MEMBER CHANGES	COLLABORATION BEFORE DEMENTIANET	CATCHMENT AREA

	AT START	END YEAR 2					

A	1 GP; 1 PN; 2 CN; 2 CM; 1 GS **(total: 7)**	1 GP; 1 PN; 2 CN; 2 CM; 1 GS **(total: 7)**	CM, GP	No	Some	Yes	Small

B	3 GP; 3 CN; 2 CM; 1 GS; 1 OT; 1 PT; 1 WF; 1 MM **(total: 13)**	2 GP; 4 CN; 2 CM; 2 GS; 2 OT; 1 PT; 1 WF; 1 MM **(total: 15)**	WF	Yes	Some	Yes	Large

C	1 GP; 1 PN; 11 CN; 1 CM; 2 GS; 4 WF; 2 MM **(total: 22)**	1 GP; 1 PN; 11 CN; 1 CM; 2 GS; 4 WF; 2 MM **(total: 22)**	GP, PN (both period absent)	No	Some	Yes	Large

D	2 GP; 5 CN; 1 CM; 2 WF **(total: 10)**	2 GP; 5 CN; 1 CM; 2 WF **(total: 10)**	CM, CN (both period absent)	No	Many	No	Large

E	2 GP; 1 PN; 2 CN; 1 CM; 1 GS; 1 WF **(total: 8)**	2 GP; 1 PN; 2 CN; 1 CM; 1 GS; 1 PH; 1 WF; 1 PT **(total: 10)**	PN, CM	No	Some	No	Small

F	2 GP; 2 PN; 1 CN; 1 CM; 1 IC **(total: 7)**	2 GP; 2 PN; 1 CN; 1 CM; 1 IC **(total: 7)**	PN, CM	No	None	No	Large

G	2 GP; 2 CN; 1 CM; 1 WF **(total: 6)**	1 GP; 2 CM; 1 WF; 1 MM **(total: 5)**	GP, CM	No	Some	Yes	Small

H	2 GP; 4 CN; 1 CM; 2 GS; 1 OT; 5 WF; 1 IC **(total: 16)**	2 GP; 3 CN; 1 CM; 2 GS; 1 OT; 1 PT; 5 WF; 1 IC **(total: 16)**	CN	No	Some	No	Large

I	1 GP; 1 PN; 1 CN; 1 CM; 1 MM **(total: 5)**	1 GP; 1 PN; 1 CN; 1 CM **(total: 4)**	CN	No	Some	No	Small

J	1 CN; 2 CM; 1 OT; 1 PH; 2 WF; 2 MM; 3 other **(total: 12)**	3 PN; 3 CN; 2 CM; 1 OT; 2 PH; 3 WF; 1 MM; 3 other **(total: 18)**	WF, OT	No	Some	No	Small

K	1 GP; 9 CN; 1 CM **(total: 11)**	1 GP; 9 CN; 3 CM **(total: 13)**	CN, CM	No	Some	No	Small

L	1 GP; 1 PN; 1 CN; 1 CM; 1 GS; 1 WF **(total: 6)**	1 GP; 1 PN; 1 CN; 1 CM; 1 GS; 1 WF **(total: 6)**	PN	No	None	No	Large

M	1 GP; 2 CN; 1 CM **(total: 4)**	1 GP; 2 CN; 1 CM **(total: 4)**	CN	No	None	No	Small

N	1 GP; 1 PN; 2 CM; 2 GS **(total: 6)**	1 GP; 1 PN; 2 CM; 2 GS **(total: 6)**	PN	No	None	Yes	Small

O	1 GP; 1 PN; 2 CN; 1 GS **(total: 5)**	2 GP; 1 PN; 1 CM; 1 GS **(total: 5)**	PN	Yes	Many	No	Large

P	1 GP; 1 POH; 2 CN; 1 CM; 1 GS; 1 WF **(total: 7)**	1 GP; 1 POH; 2 CN; 1 CM; 1 GS; 1 WF **(total: 7)**	PN	No	None	No	Large

Q	1 GP; 2 POH; 2 CN; 1 CM; 1 GS **(total: 7)**	1 GP; 2 POH; 2 CN; 1 CM; 1 GS **(total: 7)**	PN	Yes	None	No	Small


Catchment area: area from which the network attracts its population of patients with dementia, defined by geographical size and population distribution and density; large = more than approximately 7,500 persons. GP = general practitioner; PN = practice nurse; CN = community nurse; CM = case manager; GS = geriatric specialist; PH = pharmacist; OT = occupational therapist; PT = physiotherapist; WF = welfare worker; MM = management or municipality; IC = informal caregiver.

### Analyses

We calculated the mean score (range 1–4) for total network maturity to account for missing data when the interview data was not rich enough to score one of the domains. We presented descriptive data for each timepoint as means and standard deviations.

Differences in mean network maturity scores (total and sub scores) between timepoints were analysed using linear mixed models, to account for repeated measures within networks and missing data. We included random intercepts per network and a fixed effect for time. We used SPSS version 25.

#### Data integration

Network maturity scores over time were plotted in a graph and by closely inspecting these graphs, networks were identified with similar network maturity patterns. Networks with similar patterns were clustered, based on their improvement in network maturity score over time. Networks with a network maturity score above two at T2 were classified as successful, which represented a change from ad hoc to defined collaboration. Networks with a score below two at T2 were classified as unsuccessful, as their collaboration remained ad hoc after two years. By using the qualitative data from the interviews and log data we explored whether clustered networks had similar characteristics, such as existing collaboration, network size, differences in network composition or network leader(s) and leadership abilities. Moreover, by analysing the interviews we identified which positive or negative factors were important for each cluster of networks.

## Results

### DementiaNet networks

Twenty-five networks of primary dementia care professionals started with the DementiaNet program between January 2015 and April 2017. Six networks ceased active participation within the first year, reasons were either lack of intrinsic motivation (e.g. participation was initially not based on own motivation) or lack of time, resulting in insufficient momentum for a transition process. Two networks were not able to participate in the data-collection. Hence, results refer to 17 networks.

The average number of network members per network at the start was 9 (range 4–22). The average number of disciplines per network was 5 (range 3–9) at start, and 6 (range 3–10) after 2 years. Three network leaders had to stop due to sickness or change of jobs and were replaced by another network participant. A detailed description of the network characteristics can be found in ***Table 1***.

### Network Maturity

**Figure 3:**
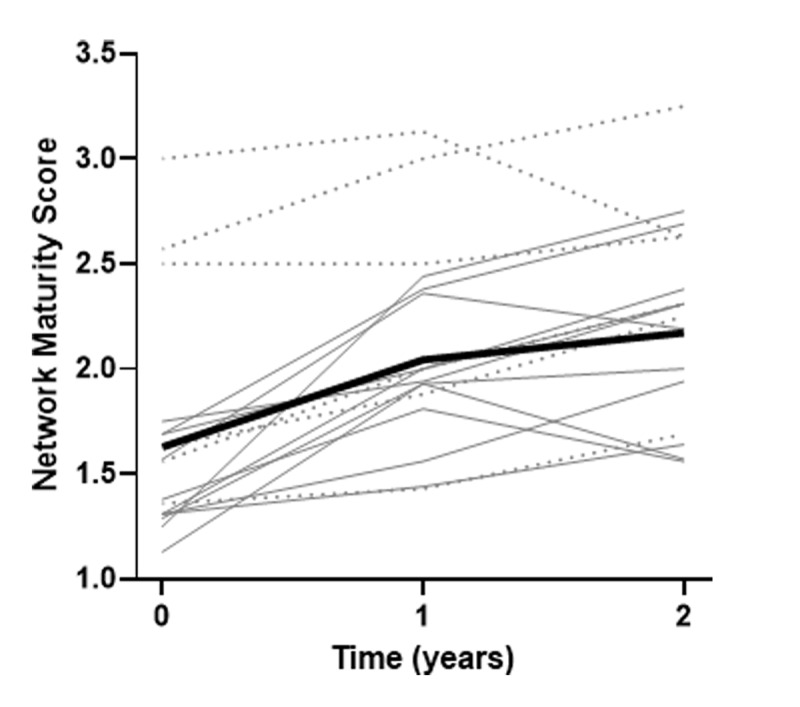
Network maturity trajectories of all networks with Network Maturity Scores on a scale of 1–4. Dashed lines represent networks with an existing collaboration, solid lines represent new networks and the bold line is the mean.

On average the networks significantly matured with a yearly increase in total network maturity score of 0.29 (95%-CI: 0.20–0.38) on a scale of 1–4, as the mean bold line graphically presents in ***Figure 3***. On average networks matured on all the integration domains except for organisational integration and system integration (***Table 2***). Network maturity domain scores increased the most for professional- and functional integration (respectively, 0.32 (95%–CI: 0.22–0.43) and 0.4 (95%-CI: 0.09–0.71)). ***Figure 3*** shows that networks with an already existing collaboration have, on average, a higher starting level and networks with a new collaboration were able to increase their network maturity the most in these two years.

**Table 2:** Total and domain specific Network Maturity scores on T0, T1 and T2 (Crude means and standard deviations; β-coefficients, 95 % confidence intervals and p-values).


	T0		T1		T2		LINEAR MIXED MODELS	

	MEAN	SD	MEAN	SD	MEAN	SD	BÈTA (95% CI)	*p*

**Total network maturity**	1.66	0.53	2.11	0.50	2.24	0.50	0.29 (0.20–0.38)	**<0.001**

**Scope**								

Person-focused care	1.38	0.57	1.72	0.60	2.06	0.66	0.27 (0.18–0.36)	**<0.001**

Population-based care	1.53	0.78	2.13	0.76	2.27	0.69	0.23 (0.13–0.33)	**<0.001**

**Type**								

Clinical integration	1.65	0.79	2.13	0.76	2.21	0.66	0.16 (0.06–0.26)	**0.003**

Professional integration	1.59	0.75	2.41	0.64	2.56	0.58	0.32 (0.22–0.43	**<0.001**

Organizational integration	1.97	0.33	2.22	0.36	2.32	0.50	0.05 (–0.01–0.11)	0.108

System integration	1.96	0.56	2.25	0.5	2.03	0.62	0.05 (–0.04–0.15)	0.246

**Enablers**								

Functional integration	1.47	0.65	1.84	0.63	2.09	0.59	0.4 (0.09–0.71)	**0.012**

Normative integration	1.82	0.68	2.25	0.66	2.44	0.68	0.18 (0.07–0.28)	**0.001**


SE = standard error, significant at p-value below 0.05, 95% CI = confidence interval.

### Network Maturity patterns

#### Successful network maturity

**Figure 4:**
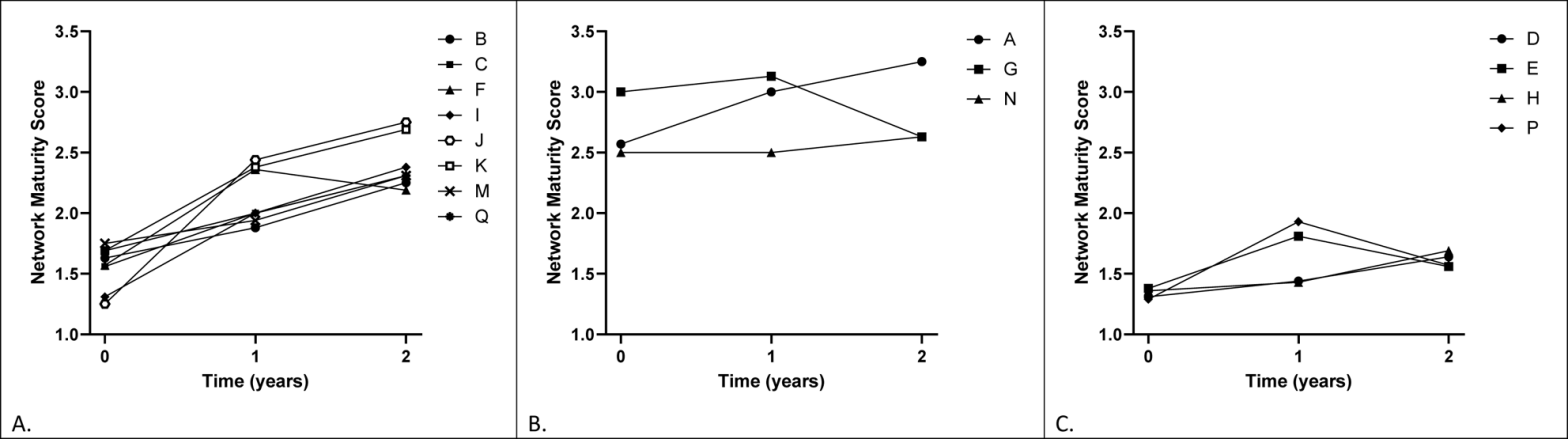
Patterns of the network maturity score trajectories over time for the networks. **A.**) Trajectories of networks where network maturity improved; **B.**) Trajectories of networks that maintained their high network maturity; and **C.**) Trajectories of networks where network maturity was not improved or sustained.

Eight networks showed a pattern of increasing network maturation that was classified as successful (see ***Figure 4A***). Based on the interviews with network leaders several facilitating factors for professional and functional integration were identified in the majority of these networks. First, these networks focused on getting familiar with each other’s expertise by organizing moments of interaction (e.g. by implementing multidisciplinary meetings) and drafting a document with everyone’s expertise and contact information. This resulted in mutual trust in the competence of the various disciplines involved. Second, this process added to more patient-related communication (e.g. by implementing a communication tool). Third, they developed work agreements regarding the population-focused view, thereby they improved their ability to identify persons with dementia in their shared population. Lastly, networks that showed successful network maturation were also characterized by an improvement in normative integration. Logs showed that network leaders’ improvement in structuring and organizing the network processes. After two years, several network leaders mentioned that their involvement had become less pronounced, due to increased commitment of the other members in the network. Maturation on organisational and system integration was more difficult to achieve, some networks explicitly mentioned that, as a local network, they have very little influence on policy development and regulations.

Three networks with relatively high network maturity scores were able to maintain the scores throughout the two-year period after initiating the DementiaNet approach (see ***Figure 4B***). These networks were pre-existent collaborations and were already matured as a network before they started with the DementiaNet program. These networks were characterized as small, experienced little changes in the team’s composition and had a clear person-focused view. The network’s stability was beneficial for the professional and normative integration. They mentioned that there was already a good foundation and serious commitment from the network members. This made it easy to work on new improvement goals. However, they indicated they were content with the current network performance, which hampered further maturation.

#### Unsuccessful network maturity

Four networks were unable to improve their network maturity score (see ***Figure 4C***). The interviews showed that this was mainly caused by factors related to normative integration: a lack of commitment from the network members and absence of general practitioner (GP) involvement. Additional reasons related to normative integration retrieved from the logs showed the absence of a capable network leader and many changes in the composition of the network. Especially when the network composition was not stable, network leaders mentioned difficulty in building trust, communicating about patient care and defining work agreements.

Most of these networks were not able to improve their scores related to person- or population focused view, because the tasks and expertise of the individual network members were unclear within the network. As a result, it was difficult, if not impossible, to define work agreements. The networks indicated that change regarding professional integration was limited. For example, it was difficult or impossible to implement multidisciplinary meetings. Furthermore, the network leaders indicated mutual respect and trust still needed to grow.

## Discussion

This study showed that the DementiaNet program seems to successfully facilitate a transition towards more mature networks, as shown by the significant yearly increase of total network maturity score of 0.29. In practice, this would mean that each year the network maturity will increase on two domains from for example ad hoc to defined collaboration. Networks with new collaborations were able to significantly improve their network maturity whereas existing collaborations maintained their already high network maturity scores. Networks showed improvement on almost all the RMIC domains except for organizational- and system integration. Professional and normative integration improved most. Facilitators to successful network maturation were ‘getting to know each other’ and building trust during the first years. Factors that could hamper successful integration were inadequate leadership, absence of active GP involvement or changes in the network composition or network leader.

The RMIC theory, that was the basis for our evaluation of network maturity development, suggests that a transition on all levels of the network (micro, meso and macro) is required to achieve complete care integration. Our study showed no significant improvements for organisational and system integration. A likely explanation it that the DementiaNet program was deliberately designed using a bottom-up approach with a focus on the local collaboration, thus the professional level. Consequently, integration mainly took place on the domains within the micro and meso level. Local networks felt, they have very little influence on policy development and regulations. This is in line with previous research, where lack of policy influence and available funds were mentioned as reasons it was difficult to include the meso and macro level in the maturation process of healthcare [31,32]. Moreover, improvements on the meso and macro level are in general very difficult to achieve [23,31,32], because of the complexity of tackling all the integration levels with an integrated care approach. Even though it is suggested that stakeholders from all levels need to be involved to achieve a sustainable collaboration [31,33], further studies are warranted to identify successful strategies to achieve this goal.

In our study, professional integration scores showed the most prominent increase. A likely explanation is that getting to know each other, building relationships and thereby trust, is crucial during the start of a network or collaboration in general [34,35,36]. Our study suggests that, thereafter, networks were able to focus on implementing work agreements related to improving populations health and improvement of their care processes. This was illustrated by the subsequent prominent increase in functional integration in our networks [37,38].

A capable network leader was deemed of major importance for the network maturation. Current literature confirms the importance of leadership for achieving care integration. Capable leaders should have relational, organizational and management skills [39]. The success of leaders increases when they are also able to involve the network and help the network members develop a sense of ownership [40].

The importance of GP involvement is to network maturity development is supported by previous research [41,42]. GPs were revealed as the most adequate leaders in integrated primary care initiatives, mainly due to the hierarchical structure and competences of GPs [39]. Moreover, within a local network a GP has a central role, because it is the gatekeeper of all the persons within a geographical area.

### Strengths and limitations

To our knowledge, it is a novelty to take an in-depth look at network maturation over time. The mixed-methods evaluation with innovative measurements and data integration strategies is an important strength of the study, as it does justice to the complexity of the DementiaNet program and local network dynamics. This approach contributed to a deeper understanding of the mechanisms behind network maturity development.

The lack of a validated measurement tool for network maturity forced us to use our own developed tool which is considered a limitation. However, the measurement tool was based on the validated RMIC and included a doubled independent and protocolized rating procedure. Interviews used for the ratings were only conducted with the network leader(s). Ideally ratings would include opinions of all network members. The missing data on the domain of system integration when data richness was lacking may have influenced the results, however the number of missing data was not substantial and we corrected for it in our analyses.

The study was carried out in the local Dutch dementia care setting, but the results are not restricted to this setting. A strength is that the tailor-made DementiaNet approach could be translated to other populations in primary care where multiple primary care professionals are involved for example in frail older adults, palliative care or other chronic conditions. This does not mean that the results of this study are also directly transferrable as different settings will have different system dynamics.

### Implications for research and practice

Whereas facilitators for network maturation identified in this study at the micro and meso level have direct practice implications (i.e. getting to know each other, building relationships and trust), achieving integration among the meso and macro level is more difficult and future research is needed to develop successful strategies [31,33] including stimulation by care organisations or the government [32,34].

For integrated care initiatives to significantly contribute to the transformation of the healthcare system, it is important that validated easy-to-use measurement tools are developed [43]. The in-depth look at network maturation over time in this study will be helpful for the development of such measurement tools. Currently, we are validating a questionnaire to measure integrated care maturation based on the RMIC domains. By using a questionnaire experiences of all the network members and also patients and informal caregivers can be taken into account.

Our study showed positive results on network maturity when using the DementiaNet approach. The DementiaNet approach is tailored to networks’ own needs and thereby reflects the variation in daily practice to a great extent. Networks can decide which educational training they want to perform and set their own improvement goals. We therefore expect that the positive outcomes of the DementiaNet approach will be sustainable when implementing this approach at a larger scale. However, future research should identify whether these changes are sustainable and if networks are able to show even more improvement. Time is necessary for networks to mature and two years is still a short timeframe [23]. Performing a long-term follow-up study to identify network maturation is therefore essential.

## Conclusion

The DementiaNet program facilitated progress towards more mature primary care networks in the first two years after inception, with diverse trajectories. Capable network leaders, GP commitment, and a stable, committed network were identified as essential factors for a successful transition towards integrated care networks. Changes in organizational and system integration appeared difficult to achieve. More focus on meso and macro level improvement strategies is required to achieve complete care integration. Our findings provide a better understanding of the mechanisms behind network maturation; future research should evaluate the sustainability of these effects and their influence on quality of primary dementia care. Such a study could profit from the development of validated instruments to measure care integration.

## Supplementary File

The supplementary file for this article can be found as follows:

10.5334/ijic.5675.s1 Appendices. Appendix I, II and III.
