# Supplementary material for: Care Integration in Primary Dementia Care Networks: A Longitudinal Mixed-Methods Study
Source: Int J Integr Care. 2021 Dec 8;21(4):29. doi: 10.5334/ijic.5675 (PMC8663750; doi:10.5334/ijic.5675)
Supplement: Appendices. — Appendix I, II and III. [file ijic-21-4-5675-s1.pdf]

APPENDIX I – Primary care in the Netherlands

Primary care for community-dwelling dementia patients in the Netherlands [44]

- Community-dwelling dementia patients receive care from multiple care professionals, including medical disciplines (primary care physician, elderly care physician), care disciplines (community nurse, case managers), and social disciplines (social workers, respite care workers).
- All Dutch inhabitants are registered at a primary care practice in close vicinity to where they live. Primary care physician referral is needed for specialist care. Indications to obtain home care are provided by municipalities or district nursing organizations.
- All Dutch inhabitants are obliged to have health care insurance and are free to choose between various private health care insurance companies. There is fragmentation in finances of services: Primary care, home care and nursing care are part of insurance and are paid for directly by private health care insurance companies; the organization and financing of social care is the responsibility of municipalities; case management is paid for by insurance companies, and exists in multiple formats and may be independent or part of home care organizations.
- Several national guidelines and documents are available on primary dementia care arrangements in the Netherlands, including guidelines for the primary care practice, a national standard for multidisciplinary dementia care, and agreements describing collaboration between the primary care practice and home care and elderly care physicians. Despite availability, uptake of and compliance with these documents in practice is low.
- Dementia care on a local level is determined by national, regional and local policies as well as existing facilities and by individual initiatives undertaken by the healthcare professionals. As a result, services and quality of local care are highly variable throughout the Netherlands.

APPENDIX II – Stepwise development of a DementiaNet network

DementiaNet networks are formed via a stepwise approach. The program for each network is tailored to the members' own needs and priorities. This tailormade approach requires the guidance of each DementiaNet team in applying the central themes. Various steps to support the network are undertaken over a 2-year period. As a wide variety of dementia care practice exists between regions, the DementiaNet approach must be adapted to local settings and needs. In some networks, team members already collaborate. Hence, these networks obviously require a different approach than those in which team members have never worked together before. In general, the following three steps are undertaken to form a network and enhance performance:

Step 1: Recruitment of network leaders. The DementiaNet team organizes training sessions comprised of interprofessional workshops that address the DementiaNet themes. DementiaNet is also promoted in various local, regional and national healthcare meetings

and through printed and online publications (1) to encourage professionals to start a network.

Step 2: Network leader forms local network. If a potential network leader is interested to join the program, the network leader and DementiaNet coordinator assess the local situation together. Detailed insight into actual dementia healthcare provision in that specific community is crucial to optimize connection to other related healthcare initiatives. If the potential network leader can organize a group of interested professionals, preferably from medical, care and social services, the DementiaNet coordinator meets with this potential team to provide information about DementiaNet and gauge support. This step usually takes3–6 months and requires the commitment of the potential network leader; it is a first test of the leadership of this individual's competencies. So far 18 network leaders have succeeded in establishing a DementiaNet network, 10 are still in the process of organizing the network and 17 healthcare professionals were not able to engage other professionals to jointly start a network.

Step 3: Implementation of the DementiaNet program. This step encompasses the implementation of the central themes, according to an action plan with: monitoring of team performance, annual self-assessment of quality of care in the local network and interprofessional and practice-based education to enhance expertise. Network leaders also join a leadership support program based on the UK National Health Service (NHS) healthcare leadership model (2). This provides individual coaching and group session workshops to improve personal leadership skills. Regular meetings facilitate long-lasting collaboration and help develop a collaborative view on healthcare (3, 4) through open discussion of task coordination and responsibilities and conflicts of interests. Prerequisites for collaboration and reflections on team performance results are also discussed in local network meetings. During the 2-year program all network members attend interprofessional training workshops, often twice a year. Network members select training topics themselves, for example on recognition of cognitive decline, dementia diagnosis, complex behavioural problems and shared decision making.

References:

1. DementieNet. Nijmegen: radboud university medical center. 2015 [Available from: https://www.dementienet.com/. [In Dutch]
2. NHS leadership academy. Healthcare Leadership Model 2016 [Available from: https://www.leadershipacademy.nhs.uk/resources/healthcare-leadership-model/.
3. D'Amour D, Goulet L, Labadie JF, Martin-Rodriguez LS, Pineault R. A model and typology of collaboration between professionals in healthcare organizations. Bmc Health Services Research. 2008;8(1):188. DOI:http://doi.org/10.1186/1472-6963-8-188.
4. Waterman H, Boaden R, Burey L, Howells B, Harvey G, Humphreys J, et al. Facilitating large-scale implementation of evidence based health care: insider accounts from a co-operative inquiry. BMC Health Serv Res. 2015;15(1):60. DOI:http://doi.org/10.1186/s12913-015-0722-6.

APPENDIX III - Topic list Network Maturity DementiaNet

RMIC domain	Topics

Professional integration	- Tasks and expertise's<br>- Respect and trust
Clinical integration	- Work agreements<br>- Multidisciplinary care plan and meeting
Normative integration	- Goal<br>- Commitment network members<br>- Leadership
Person focused care	- Person-centredness
Population base care	- Early signalling<br>- Coordinator for persons with dementia
Functional integration	- Digital systems<br>- Feedback
Organisational integration	- Support from organization
System integration	- Policy developments
